# Using Deep Learning for Task and Tremor Type Classification in People with Parkinson’s Disease

**DOI:** 10.3390/s22197322

**Published:** 2022-09-27

**Authors:** Ghazal Farhani, Yue Zhou, Mary E. Jenkins, Michael D. Naish, Ana Luisa Trejos

**Affiliations:** 1 Department of Electrical and Computer Engineering, Western University, London, ON N6A 5B9, Canada; 2 School of Biomedical Engineering, Western University, London, ON N6A 5B9, Canada; 3 Movement Disorders Program, Clinical Neurological Sciences, Western University, London, ON N6A 3K7, Canada; 4 Department of Mechanical and Materials Engineering, Western University, London, ON N6A 5B9, Canada

**Keywords:** Parkinson’s hand tremors, classification of hand tremor types, deep learning

## Abstract

Hand tremor is one of the dominating symptoms of Parkinson’s disease (PD), which significantly limits activities of daily living. Along with medications, wearable devices have been proposed to suppress tremor. However, suppressing tremor without interfering with voluntary motion remains challenging and improvements are needed. The main goal of this work was to design algorithms for the automatic identification of the tremor type and voluntary motions, using only surface electromyography (sEMG) data. Towards this goal, a bidirectional long short-term memory (BiLSTM) algorithm was implemented that uses sEMG data to identify the motion and tremor type of people living with PD when performing a task. Moreover, in order to automate the training process, hyperparamter selection was performed using a regularized evolutionary algorithm. The results show that the accuracy of task classification among 15 people living with PD was 84±8%, and the accuracy of tremor classification was 88±5%. Both models performed significantly above chance levels (20% and 33% for task and tremor classification, respectively). Thus, it was concluded that the trained models, based on using purely sEMG signals, could successfully identify the task and tremor types.

## 1. Introduction

Parkinson’s disease (PD) is the most common neurodegenerative movement disorder, affecting 2–3% of the senior population (age ≥ 65 years) [1]. The most prominent motor symptoms of PD are tremor (i.e., involuntary movement of body parts), rigidity (i.e., resistance to externally imposed movements), bradykinesia (i.e., slowness of movement), and postural instability [2]. As the pathological hallmark of PD is the loss of dopaminergic neurons in the substantia nigra pars compacta, the current treatment of PD is based on the replacement of dopamine. The available treatments can reduce the motor symptoms; however, due to the fluctuations of dopamine levels in the brain, the severity of the symptoms can drastically fluctuate and negatively influence the lives of people living with PD (PwP) [3].

Among PD symptoms, hand tremor is very common. It is estimated that between 79% and 90% of PwP suffer from hand tremors [2]. The two main classes of tremors are rest tremor and action tremor. Rest tremor occurs when relevant muscles are at rest, while action tremor occurs when relevant muscles are activated. Action tremor includes postural, kinetic, intention and specific tremor [4,5]. For many PwP, hand tremor is highly disabling and treatment is needed. Even though a few medications are available, no solid evidence exists regarding the effectiveness of most recommended treatments [6,7]. Thus, the treatment of hand tremors remains limited, such that one-third of patients discontinue taking their tremor medications [8]. Hence, alternative methods to assess and manage the tremor should be considered. Recently, wearable robotic systems have shown promising results in providing rehabilitation and assistance in movement problems [9]. Wearable devices have shown high accuracy in assessing the severity of tremor [10]. However, tremor management can be more challenging. For example, the mechanical suppression of tremor has been used to improve voluntary motions [11]. However, as the time evolution of tremor follows non-linear dynamics and can significantly vary from one person to another [12], modeling tremor and distinguishing the voluntary motion from tremor can be challenging. Moreover, most of the current approaches are focused on predicting the tremor [13,14]. However, strong evidence of the existence of low dimensional chaotic structures in the dynamics of cortical activity of PwP has been observed [15]. The consequence of dealing with a chaotic system is that even infinitesimal errors in the initial condition of a predictor model will grow exponentially fast. Consequently, the prediction horizon of tremor estimation is extremely limited in time. An alternative solution was proposed by Taheri et al. [11], in which instead of estimating tremor, the muscle torque that produced the tremor was estimated, and then an equal and opposite torque would be applied to suppress tremor. The main challenge of this approach is the inability to attenuate tremor without interfering with voluntary motion. Hence, as motion of PwP are combinations of their voluntary movements and tremor, it is essential that the voluntary movement not be taken as tremor [16]. Thus, identifying the task (gesture) and the tremor type (resting, postural, or action) can provide crucial information for a control system. Furthermore, as many PwP experience muscle weakness [17], they require assistance with their voluntary movements, thus identifying the voluntary movement is deemed necessary.

Currently, surface electromyography (sEMG) sensors are one of the most common technologies that are used for controlling wearable devices or for hand gesture recognition [18]. For example, in the rehabilitation sciences, sEMG is frequently used to examine voluntary muscle contractions [19]. In sports physiology, sEMG signals are routinely used to coordinate the movement optimization and estimate muscle fatigue [20]. The possibility of using sEMG to study muscle behaviours has been investigated in the field of clinical neurophysiology [21,22]. Moreover, sEMG has proven beneficial in differentiating between muscle movement characteristics of PD and healthy motion [23,24,25]. Although, sEMG data have shown promising results in the mentioned research areas, their potential to identify parkinsonian hand tremor type and gesture classification has remained understudied. For example, in a small study with only one PwP participant, sEMG detectors were placed on specific muscles to determine their voluntarily movements [26]. The participant was not performing any real task, and they just moved their hand to engage the target muscle. Investigating the possibility of using sEMG data to identify voluntary hand motions during task performance can be extremely important to help PwP with task performance and to suppress tremor.

In many wearable robotic tremor suppression devices, IMUs are being used [27,28,29]. Alternatively, using sEMG data means that parts of the sensing system can be moved from the hands onto the forearms to reduce the required hardware within the device. Furthermore, as the orientation of the electrode does not change the output data, the calibration process of the tremor suppression devices could be simpler, and the devices could become more user friendly.

The contribution of this work is that it is the first implementation of a machine learning (ML) algorithm that can successfully identify motions of PwP, as well as the tremor type, purely from sEMG signals. When performing a task in the presence of tremor, accurate identification of the voluntary motions and distinguishing the type of tremor can significantly improve the design of wearable robotic tremor suppression devices, which could lead to improving the lives of PwP. Recently, ML methods have been used for both the identification and prediction of bio signal from sensors. As these algorithms have been shown to be able to find patterns in complex data, they were selected as the main statistical tool to identify the tremor types as well as intended voluntary motions of the people in the current study. A brief review of the proposed ML method is presented in Section 2. Section 3 provides details about the experimental setup for the training of the ML algorithms. In Section 4, the results of the study are shared, and Section 5 provides a discussion related to the results of the study.

## 2. Background

Deep learning algorithms have shown promising results in biomedical fields, and they have been vastly implemented in the design of human assistive devices [30,31,32,33]. In biosignal data classification and prediction, as the time sequence of actions is important (the most recent time steps have the highest predictability), algorithms that are sequence-aware are highly desired. The bidirectional long short-term memory (BiLSTM) algorithm is one of the popular deep learning algorithms with a specific network architecture designed to handle sequences of data [34]. In the present study, the BiLSTM algorithm was employed.

### 2.1. BiLSTM

The structure of a conventional LSTM is designed with special memory cells that are able to store data temporarily. The network contains three gates known as the input gate, the forget gate, and the output gate. In the forward direction, for input vector $x_{t}$, a memory block at time step *t* is defined as follows:

(1) $$\begin{array}{cc} i_{t} & =\sigma (W_{i}x_{t}+h_{t-1}), \end{array}$$

(2) $$\begin{array}{cc} f_{t} & =\sigma (W_{f}x_{t}+h_{t-1}), \end{array}$$

(3) $$\begin{array}{cc} o_{t} & =\sigma (W_{o}x_{t}+h_{t-1}), \end{array}$$

(4) $$\begin{array}{cc} c_{t} & =f_{t}⊙c_{t-1}+i_{t}⊙\tanh\left(W_{c}x_{t}\right), \end{array}$$

(5) $$\begin{array}{cc} h_{t} & =\tanh\left(c_{t}\right)⊙o_{t}, \end{array}$$

where σ is a nonlinear activation function, W is the matrix of parameters that are being optimized, and $i_{t}$, $f_{t}$, $o_{t}$, and $c_{t}$ are the input, forget, cell, and output gates. The element-wise multiplication is shown by ⊙. $h_{t-1}$ and $h_{t}$ are the hidden layers at time step t−1 and *t*, respectively.

In the BiLSTM, the information from the past and future of the current time frame is fed as input (two time directions). Training occurs simultaneously in the forward (positive) and backward (negative) time directions. The output sequence ($\vec{h}$) of the forward LSTM is obtained in the same way as the unidirectional (conventional) model. The output sequence of the backward LSTM is calculated as the input data are fed into the model in reverse order. The BiLSTM architecture learns long-term dependencies in a time series, and in many studies, the algorithm has outperformed the conventional LSTM [35,36].

### 2.2. Neural Network Architecture

One crucial aspect of training a BiLSTM model, or any deep learning algorithm, is the choice of neural architecture. Manual development of optimized architectures by human experts is quite common. However, the approach is very time consuming for researchers and prone to error. There is a growing interest in implementing automated methods for the neural architecture search (NAS). Several algorithms for NAS have been proposed in the literature [37]. Among these, the biologically inspired evolutionary algorithms (EA) have been proven to be efficient in finding architectures [38]. Typically in EAs, a group of individual models are called a population, and the size of population *p* is fixed. The models within a population are initialized based on random architectures and then improved iteratively. At each cycle, *k* random models are selected from the population (k<p). The fitness of each of these models is evaluated and the one with the highest fitness score is selected as the parent. Then, through a mutation, which is a small and random modification of the parent architecture, a child architecture is constructed. The child model is trained, evaluated, and then added to the population. Typically, at the end of each cycle, the model with the lowest fitness score is killed. The process is called tournament selection [39]. Recently, Real et al. [40] introduced the aging evolution approach that eliminates the oldest model in a population rather than killing the least fit model in the tournament selection. The aging evolution favors the newer models, thus instead of a premature focus on good models, more architecture choices will be explored. Compared to the non-aging evolution, the aging evolution resulted in better outcomes, yet retained a high efficiency. Thus, the latter approach has been chosen for NAS in the present work.

The evolutionary algorithms are based on evaluating models within a population by running several cycles, and users determine how many cycles are needed. Several methods have been proposed to indicate the termination criterion [41]. In the present work, the best-worst termination criterion was adopted. In this approach the algorithm stops when the difference between the best and worst objective values of the population is less than a predefined threshold [42].

### 2.3. Warm Initialization

During the training of a neural network (NN) model, the parameters W of the model are optimized. Typically, at the first step, weights W are randomly initialized, and as the training proceeds, they converge to the optimized solution. Machine learning models are not static, and as new data are acquired, the models need to be retrained. However, for similar datasets, it is expected that the models will be similar. Thus, for similar datasets, it is natural to initialize the new parameters using parameters of the already trained model. In this approach, instead of giving a fresh parameter initialization, a “warm-start” initialization is used [43]. Some studies have shown that the warm initialization can accelerate the optimization process [44]. In the present work, the impact of warm initialization on the accuracy of the models was investigated.

## 3. Materials and Methods

### 3.1. Data Collection

A movement disorders neurologist collected data from 17 PwP in the Wearable Biomechatronics Laboratory at Western University. The study was approved by the Health Sciences Research Ethics Board (#106172). Using an eight-channel sEMG sensing device (Myo, Thalmic Labs), data from the forearm with the dominant tremor were collected at a sampling rate of 200 Hz. The 4th channel of the Myo armband was aligned with the longitudinal axis of the Extensor Carpi Ulnaris. A detailed explanation of the alignment of the Myo Armband’s electrodes to muscles can be found in [45]. To eliminate the motion from other parts of their bodies, the forearm of each participant was securely strapped to the table such that only the hand could freely move in space. The focus of the present study was to assess the three types of tremor: resting, postural, and action tremor. Resting tremor occurs when the person’s hands are at rest, postural tremor happens when PwP attempt to maintain a hand position against gravity, and action tremor is observed during voluntary hand movements. Participants recruited for the study included those for which their current treatment was not effective in managing their tremor. To assess these three tremors, the participants were asked to perform six different tasks as follows [46]:

**Task** **1a:** The participant was asked to rest their hand while the palm was facing down.
**Task** **1b:** The participant was asked to rest their hand while the palm was facing up.
**Task** **2:** The participant was asked to hold their hand in a postural position.
**Task** **3:** The participant was asked to move from a flexion position to an extension position, pinch a pencil, and then move back to the flexion position.
**Task** **4:** The participant was asked to move a pencil with the thumb and index finger, while they were extending the wrist joint.
**Task** **5:** The participant was asked to draw a spiral.

It was of interest to classify the tremor types as well as identify some general voluntary muscle motions. Tasks 1a and 1b were chosen as they are the typical resting hand postures. Task 2 represents a common case of a postural hand gesture. Tasks 3, 4, and 5 were chosen as they involved flexion and extension of the wrist while performing an action, thus the action tremor type could be assessed. Furthermore, Tasks 3 and 4 represent some of the most important tasks, such as grabbing objects, and Task 5 was a simplified version of a writing task.

Tasks 1a, 1b, and 2 were recorded for one minute. Tasks 3 and 4 were repeated five times, and Task 5 was performed once, while participants were performing the six tasks, the motions of their hands were video recorded. The initial assessment of videos, showed that two participants had difficulties performing some of the tasks. They were unable to hold their hands in a postural position, and they had difficulties pinching and moving a pencil. Thus, the data from these two participants were discarded. The analysis was performed on the remaining 15 participants, nine were male and six were female. The average age of the participants was 69 and they were all taking medication to treat the symptoms of PD.

### 3.2. Data Preprocessing

Data processing was done using the SciPy and NumPy libraries in the Python programming language. Using Scipy built-in functions, the collected raw data were filtered with a 4th-order Butterworth low-pass filter with a 20 Hz cut-off frequency. The Myo armband has a sampling frequency of 200 Hz. Based on the Nyquist theorem the maximum frequency contained in the recorded sEMG signals is 100 Hz. However, for identifying the muscle activities corresponding to the hand and arm motions of PwP, a lower frequency cutoff has been suggested in previous studies [47,48,49] and adopted herein.

After implementing the filter, the overlapped windowing technique was applied to generate data segments that were 250 ms in length. Longer windows lengths were also examined, however, they did not improve the classification results. In the present work, the analysis and testing of the model were done offline. Of note, considering that the processing time to provide the classification result in real-time systems are typically less than 300 ms [50,51], a window length of 250 ms facilitates any future real-time analysis. To increase the number of segments used as input data, the windows were overlapped by 50%. Finally, the measured sEMG data were fed into the deep learning algorithm as the matrix $S=(S_{1}^{l},S_{2}^{l},\ldots ,S_{8}^{l})$, where $S_{i},i\in \left[1,8\right]$ represents each of the sEMG channels, and *l* represents the length of the window. The models were subject-specific, thus each participant had a personalized model trained based on their data. Data from each participant were split into train and test data. In biosignal analysis, it is a common practice to train an algorithm on the historical data and test on the more recent sequences [52,53,54]. The sEMG data represent a time series, hence the first segment of data (80% of the total) was used for training, and the final 20% was used for the test.

### 3.3. Neural Architecture Search

In NNs, hyperparameter tuning is an important yet challenging task. Often, human experts determine the best configuration of hyperparameters based on trial and error. As mentioned in Section 2.2, an automatic search for an optimal neural architecture is an appropriate alternative to the trial and error attempts. In the current study, the hyperparameter tuning process was automated using the regularized evolutionary algorithm [40]. To implement the algorithm, sample Python codes that were shared by Real et al. [40] were modified and used for NAS.

The experiments were conducted choosing from the stochastic gradient descent (SGD) and Adam algorithms. Many studies have shown that training via Adam could be much faster and more accurate, while others have suggested that training via SGD performed better on the validation data [55,56,57,58]. Thus, it was of interest to examine which of the two popular optimization methods had better performance for the data in the present study. Furthermore, the number of neurons per layer, the learning rate, the batch size, and the choice of the activation function were also selected for tuning. The complete list of parameters, as well as the range of available choices for each parameter, are shown in Table 1. Of note, the neural architecture search was performed using the data for each individual. Thus, each participant had a personalized set of hyperparameters.

### 3.4. Warm Initialization

**Figure 1:**
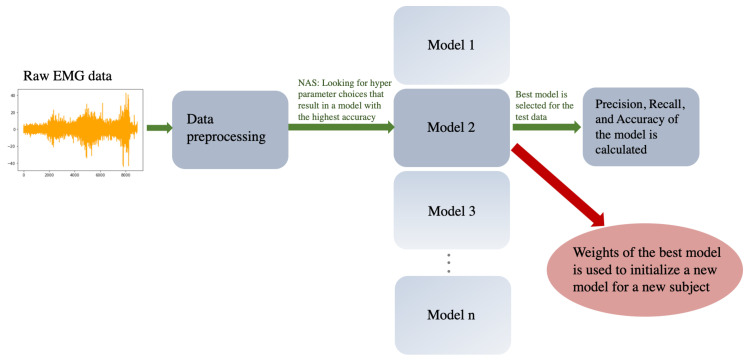
Flow chart of all major steps required from pre-processing the raw data to evaluating the results.

As the models were subject-specific, the training process was repeated for each participant. To investigate whether the warm initialization could improve the accuracy of the trained models after a model was trained for a participant, the weights of the trained model were used to initialize the weights of a new model for another participant. Then, for each participant, the accuracy, precision, and recall of the trained models based on warm initialization, and those based on random initialization of the weights were calculated and compared to each other. The general schematic outlining the steps that need to be taken to train the model is summarized in Figure 1.

### 3.5. Evaluation of Models

To evaluate the performance of the classifiers, the confusion matrix was used. A confusion matrix can provide the following information:

**True positives** **(TP):** number of tasks that are correctly labeled as a specific task
**False positives** **(FP):** number of tasks that are incorrectly labeled as a specific task
**True negatives** **(TN):** number of tasks that are correctly labeled as not belonging to a specific task
**False negatives** **(FN):** number of task that are incorrectly labeled as not belonging to a specific task.

To further evaluate a model, the accuracy, precision, and recall can be calculated. They are defined as follows.

$$\begin{array}{cc} \mathrm{Accuracy} & =\frac{\mathrm{TP}+\mathrm{TN}}{\mathrm{TP}+\mathrm{TN}+\mathrm{FP}+\mathrm{FN}}\\ \mathrm{Precision} & =\frac{\mathrm{TP}}{\mathrm{TP}+\mathrm{FP}}\\ \mathrm{Recall} & =\frac{\mathrm{TP}}{\mathrm{TP}+\mathrm{FN}} \end{array}$$

These metrics were used to evaluate the models for each task.

## 4. Results

The focus of the current study was to investigate the possibility of using sEMG data to classify tasks in the presence of hand tremor, as well as to classify the tremor type. Thus, two independent models were trained. Results for each of the two models are presented in this section.

### 4.1. Task Classification

To start, a first model was built to identify which task was being performed among the six possible tasks. However, the trained models often mixed up Tasks 3 and 4, resulting in an average precision for Tasks 3 and 4 for all participants of 54±15% and 64±16%, respectively. The average accuracy was 72±6%. As an example, the confusion matrix of Participant 5 is shown in Table 2. Although, the trained model could successfully identify Tasks 1a, 1b, 2, and 5, it had difficulties distinguishing between Tasks 3 and 4. Compared to other tasks, the precision for Tasks 3 and 4 were also about 60%, significantly lower compared to the precision of other tasks (Table 3). The overall accuracy of the classifier for Participant 5 was 79%.

The overall accuracy of 72% for a 6-class classification model was much higher than a chance level of 16.6% (representing the chance level of each class). However, Tasks 3 and 4 were very similar to each other and they involved the same muscle activities. As sEMG is sensitive to muscle activation, the recorded data from the two tasks was insufficient to train the model to differentiate them. The low accuracy of the model for Tasks 3 and 4 was not due to insufficiency of the learning algorithm (as the algorithm has much higher performance for other tasks) and cannot simply be fixed by changing the placement of the armband, as the two tasks involved same motions. Thus, it was decided to combine Tasks 3 and 4 into one single label, indicating any motion related to the extension of the wrist joint, and pinching of a pencil. Subsequently, the new models based on 5 labels were trained. The result of training a model based on the 5 label classification for Participant 5 is presented in Table 4. Moreover, the mean accuracy of trained models for all subjects based on the 5 class classification was 84±8%. The precision and recall for each task are also shown in Table 5. The precision and recall for each task is at least 78%, indicating that the BiLSTM algorithm performs well. The standard deviation of precision and recall for Task 1b and Task 5 are higher than other tasks. For some participants, Task 1b was confused with Task 1a. Considering that both tasks are the resting positions of the hand (one of them indicates palm up and the other palm down posture) the confusion between the two tasks is not surprising. Task 5 indicates that the circle drawing motion also had a higher standard deviation among participants. This is because while some participants were attempting to draw circles, they moved their hands up and down, or they paused for some time, resulting in the model confusing their motion with the other motions. Although these deviations were not observed in most participants, considering the small size of the dataset, it resulted in observing a higher standard deviation for Task 5.

### 4.2. Tremor Classification

Separate subject-specific models were trained for the tremor classification into resting, postural, and action tremors (3-class classifier). The average accuracy among all participants stood at 88±5%. The precision and recall for each tremor type are shown in Table 6.

### 4.3. Warm Initialization

Repeating the training for both tremor and task classification models using the warm initialization method did not improve the overall accuracy of the models. However, in task classification, for two participants, an improvement of precision and recall for one or two tasks was observed. For Participant 1, the precision of Task 1a increased from 50% to 70%. For Participant 6, the precision and recall of Task 1a and 1b were improved. The precision and recall of Participant 6 using the random initialization as well as the warm initialization are shown in Table 7.

## 5. Discussion

The focus of this study was to investigate if low-cost sEMG devices could be used with deep learning models to identify hand tremor types in PwP, as well as the voluntary motions performed. In the present study for tremor type classification and voluntary motion identification, a short sequence of data was used and fed into a network. Thus, a natural choice for the ML algorithm was a BiLSTM algorithm that is a sequence-aware algorithm. As a result of employing a BiLSTM algorithm, crucial information related to the time sequence of motions was conserved. The accuracy of task classification stood at 84±8%, and the accuracy of tremor classification was 88±5%. Of note, both models performed significantly above the chance levels (20% and 33% for task and tremor classification, respectively) which indicates that the models could successfully identify the task and tremor types. The results of the study clearly indicate that sEMG signals can be used to identify voluntary motions and thus they can be used in tremor suppression devices. This study was also the first attempt to use sEMG data to identify PwP voluntary motions during the performance of daily tasks (such as grabbing objects). The potential of using sEMG for identifying voluntary motions has been understudied. The only similar study was conducted on a single participant and rather than performing common tasks, the participant was performing eight movements targeting each muscle individually. Thus, their result could not be generalized to PwP performing activities of daily living, such as grabbing objects or drawing. Furthermore, the results from a single participant could not be extrapolated to a larger population. Although the present study is based on a small data set, it could still be used to provide an analysis of the accuracy of the trained models among the participants.

Moreover, using the warm initialization approach, the average accuracy of the model among the participants remained the same. Modest improvements in a few tasks were observed for only two participants; therefore, no conclusive statements related to the benefits of using the warm initialization approach can be presented. Nevertheless, as the warm-start could be closer to the optimal solution [44], it would be a better initialization option. Thus, the warm initialization remained part of the proposed algorithm.

Another focus of the present study was to automate the hyperparameter tuning process. Hyperparameter tuning is an important yet challenging task that typically requires hours of time from an expert. In this paper, by implementing a regularized evolutionary algorithm, the search for the optimal neural architecture became fully automated. Thus, several parameters, including the number of neurons, learning rate, number of epochs, batch size, activation function, and optimization algorithms, were considered, and the best neural architecture for each subject was built. This can be an important step toward building practical tremor suppression devices, as the optimal hyperparameters can be found automatically, and accurate models can be trained.

The present study provides proof that the BiLSTM algorithm can identify a user’s tremor type, as well as their intended motion, from sEMG data alone. However, the experiments were limited to a few minutes of data, which was one of the main limitations of the current study. One of the major goals of implementing personalized ML models is to adapt the tremor suppression devices to the progression of the disease. Thus, going forward, it would be worthwhile to expand the duration of the data collection experiments to several hours or even several days, so that all possible motion and pattern variations of PwP can be recorded and fed into a classification model. Having access to longer periods of data would make it possible to examine the accuracy of models trained based on earlier data, and to explore the extent of accuracy in time. For example, having access to data collected every minute during a month would allow an examination would allow an examination into whether training the models based on the first week of data would result in accurate classifications in the last few days of the month, that would allow an assessment of whether the generalization holds over time and over more people. Another important aspect of conducting longer experiments is that the model could be trained and evaluated based on data collected outside of the laboratory. As reported in other studies, models trained using outside data had higher uncertainties [59], thus it is important to evaluate the models under more realistic circumstances than within a lab setting. Of note, in the present study, the number of participants was limited to 15, thus the average accuracy among all of the participants had a higher standard deviation. One future direction is to conduct the experiment with a larger population of participants, as it is expected that repeating the experiment with an increased number of participants and longer duration experiments will minimize the effect of outliers.

It is also worth mentioning that the recruitment of participants for the current study was limited to PwP attending the local clinic, thus recruiting an equal number of males and females was challenging. The participants included nine male and six female participants, which was a balanced representation of the population, as Parkinson’s Disease affects more males than females. Of note, the evaluations did not indicate that the sex of the patients had a statistical effect on the results.

Furthermore, the present study used sEMG data to train the model to investigate whether the sEMG data alone could be used for task and tremor classification. Since the IMU data for the experiments were also recorded, it would be interesting in future work to train models using the IMU data, and to compare the accuracy of ML models that are trained using each of the data sets and their combination. Finally, another direction for future work will explore embedding this algorithm into the control system of a wearable device, and evaluate the accuracy of classifications in real time.

## 6. Conclusions

To summarize, the main contribution of this work was to explore whether the proposed BiLSTM algorithm could identify the tremor type as well as the motion of PwP while performing a task. Among 15 PwP, the accuracy of task classification stood at 84±8% and the accuracy of tremor classification was 88±5%. Thus, the present study indicates that, by the use of a proper ML algorithm, sEMG data are capable of identifying tremor types. Although, the immediate implementation of the algorithm into wearable tremor suppression devices was not discussed in the paper, considering that the total processing time was less than 300 ms (data were segmented into 250 ms long windows and the data pre-processing time is less than 50 ms), the proposed model could potentially be implemented within a real-time suppression system.

## Figures and Tables

**Table 1:** Hyperparameters that were selected for optimization.

Hyperparameter	Available Options
Number of Neurons	20, 50, 100, 200, 500
Learning Rate	1 × 10${}^{-3}$, 1 × 10${}^{-4}$, …, 1 × 10${}^{-8}$
Number of Epochs	10, 20, 50, 100, 250
Batch Size	32, 64, 128, 256
Optimizer	Adam, SGD
Activation Function	tanh, ReLU

**Table 2:** Confusion matrix for Participant 5, based on the 6 label classification.

Task 1a	190	39	0	6	31	20
Task 1b	4	308	0	0	0	0
Task 2	0	0	258	0	4	0
Task 3	4	0	23	172	88	21
Task 4	2	5	5	76	216	3
Task 5	0	4	0	20	21	233

**Table 3:** Precision and recall for Participant 5, based on the 6 label classification.

Task Type	Precision	Recall
Task 1a	0.95	0.71
Task 1b	0.87	0.99
Task 2	0.90	0.98
Task 3	0.63	0.56
Task 4	0.60	0.70
Task 5	0.90	0.83

**Table 4:** Confusion matrix for Participant 5, based on the 5 label classification.

Task 1a	249	14	0	3	0
Task 1b	1	307	0	2	2
Task 2	0	0	259	1	2
Task 3 or 4	5	5	12	553	39
Task 5	0	0	0	23	246

**Table 5:** Average precision and recall of all participants, based on the 5 label classification.

Task Type	Precision	Recall
Task 1a	0.95 ± 0.06	0.71 ± 0.10
Task 1b	0.78 ± 0.15	0.99 ± 0.18
Task 2	0.92 ± 0.05	0.98 ± 0.08
Task 3 and 4 (combined)	0.82 ± 0.07	0.70 ± 0.06
Task 5	0.82 ± 0.12	0.83 ± 0.20

**Table 6:** The average precision and recall of all participants, for the tremor classification model.

Tremor Type	Precision	Recall
Resting	0.86 ± 0.08	0.92 ± 0.05
Postural	0.90 ± 0.05	0.87 ± 0.08
Action	0.92 ± 0.05	0.88 ± 0.04

**Table 7:** The comparison of precision and recall of the trained model based on random initialization and warm initialization for Participant 6.

Task Type	Random Initialization		Warm Initialization	
	Precision	Recall	Precision	Recall
Task 1a	0.60	1.00	0.81	1.00
Task 1b	0.76	0.41	0.92	0.83
Task2	0.99	1.00	1.00	1.00
Task 3 and 4 (combined)	0.87	0.88	0.89	0.94
Task 5	1.00	0.71	1.00	0.76

## Data Availability

The data obtained in this study have not been approved by the Research Ethics Board for open access and are therefore not available to the public.
